# Intraindividual time-varying dynamic network of affects: linear autoregressive mixed-effects models for ecological momentary assessment

**DOI:** 10.3389/fpsyt.2024.1213863

**Published:** 2024-03-20

**Authors:** Shakoor Pooseh, Raffael Kalisch, Göran Köber, Harald Binder, Jens Timmer

**Affiliations:** ^1^Center for Interdisciplinary Digital Sciences (CIDS), Technische Universität Dresden, Dresden, Germany; ^2^Freiburg Center for Data Analysis and Modeling (FDM), Institute of Physics, University of Freiburg, Freiburg, Germany; ^3^Leibniz Institute for Resilience Research (LIR), Mainz, Germany; ^4^Neuroimaging Center (NIC), Focus Program Translational Neuroscience (FTN), Johannes Gutenberg University Medical Center, Mainz, Germany; ^5^Institute of Medical Biometry and Statistics (IMBI), Faculty of Medicine and Medical Center, University of Freiburg, Freiburg, Germany; ^6^CIBSS-Centre for Integrative Biological Signalling Studies, University of Freiburg, Freiburg, Germany

**Keywords:** affect dynamics, mood networks, ecological momentary assessment, mixed-effects models, autoregressive models

## Abstract

An interesting recent development in emotion research and clinical psychology is the discovery that affective states can be modeled as a network of temporally interacting moods or emotions. Additionally, external factors like stressors or treatments can influence the mood network by amplifying or dampening the activation of specific moods. Researchers have turned to multilevel autoregressive models to fit these affective networks using intensive longitudinal data gathered through ecological momentary assessment. Nonetheless, a more comprehensive examination of the performance of such models is warranted. In our study, we focus on simple directed intraindividual networks consisting of two interconnected mood nodes that mutually enhance or dampen each other. We also introduce a node representing external factors that affect both mood nodes unidirectionally. Importantly, we disregard the potential effects of a current mood/emotion on the perception of external factors. We then formalize the mathematical representation of such networks by exogenous linear autoregressive mixed-effects models. In this representation, the autoregressive coefficients signify the interactions between moods, while external factors are incorporated as exogenous covariates. We let the autoregressive and exogenous coefficients in the model have fixed and random components. Depending on the analysis, this leads to networks with variable structures over reasonable time units, such as days or weeks, which are captured by the variability of random effects. Furthermore, the fixed-effects parameters encapsulate a subject-specific network structure. Leveraging the well-established theoretical and computational foundation of linear mixed-effects models, we transform the autoregressive formulation to a classical one and utilize the existing methods and tools. To validate our approach, we perform simulations assuming our model as the true data-generating process. By manipulating a predefined set of parameters, we investigate the reliability and feasibility of our approach across varying numbers of observations, levels of noise intensity, compliance rates, and scalability to higher dimensions. Our findings underscore the challenges associated with estimating individualized parameters in the context of common longitudinal designs, where the required number of observations may often be unattainable. Moreover, our study highlights the sensitivity of autoregressive mixed-effect models to noise levels and the difficulty of scaling due to the substantial number of parameters.

## 1 Introduction

Recent developments in emotion research and clinical psychology have highlighted the substantial impact of emotional fluctuations on an individual’s daily experiences, behaviors, and decisions (1). This recognition has led to the conceptualization of the evolution of affective states throughout daily life as complex networks comprising interacting emotional or mood variables. Affective network models may shed light on general rules underlying the generation and regulation of moods and on individual differences therein. Moreover, when integrated with other variables, affective networks may also elucidate the relationship of moods to other aspects of human behavior or their importance for longer-term outcomes, such as psychological well-being or functioning (2–4).

Network models have also found utility in investigating the dynamics of mental states in other areas, most prominently in clinical psychology and psychiatry. In these fields, mental disorders have been conceptualized as networks of symptoms (e.g., hypervigilance, despair, or anhedonia) that interact via biological, psychological, and social mechanisms (5–7). Although both cases of network models use the same principles, in terms of modeling on an abstract level, they live on different time scales. Moods or emotions might change quickly over short time intervals and several times a day, whereas the definition of a symptom per se usually requires its presence over at least a few weeks. In this work, we focus on network models for moods and will use the extensively studied case of symptom networks for comparison and reference, to thus better highlight specific requirements of mood/emotion modeling.

The dynamics of these networks can be modeled using time-series data. This has been facilitated by technological improvements in recent years and the fact that smartphones and wearable devices have become inseparable from the daily lives of many people. It is now a well-established practice to acquire data by experience sampling methods (8), where individuals are observed frequently within short periods of time, e.g., several times a day over 1 to 2 weeks, or once every day for a couple of weeks, resulting in the so-called ecological momentary assessment (EMA) data. As the acquisition of EMA data is made easier, single-subject analysis is also gaining momentum, where constructing individual dynamic networks might be helpful in understanding the mental states of a specific person, and this, in turn, might promote personal prediction or also personal interventions (9, 10).

**Figure 1:**
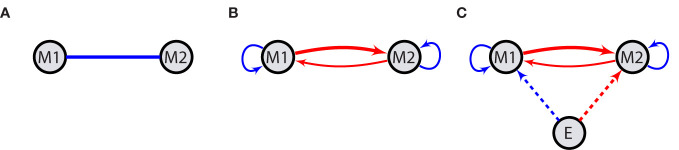
Network structure. Emotions or moods, M1 and M2, interact with each other leading to meaningful contemporaneous correlation between them. This is shown by a solid line **(A)**. Solid arrows represent temporal correlations meaning that the value of the source variable (node) at time *t* has an impact on the value of its target at time *t* + 1. Looped arrows indicate autoregressive effects **(B)**. Exogenous factors E act on moods at the same time in a one-way fashion **(C)**. Blue (dark gray) and red (light gray) arrows indicate activation and suppression, respectively. Different thicknesses stand for distinct interaction strengths. Dashed arrows indicate contemporaneous effects, while solid ones are indicative of possible temporal causalities.

In constructing such networks, where one variable (observed mood) is correlated to the other, the corresponding network nodes (M1 and M2 in **Figure 1**) can be seen as being connected with a weight according to the type (positive or negative) and the intensity of the correlation (line in **Figure 1A**). For instance, in the case of moods, a cheerful mood may frequently co-occur with a satisfied mood and be inversely related to irritated, anxious, or sad mood states. A connection may also be of some causal nature such that one variable, M2, at time *t* + 1 is highly correlated (positively or negatively) with the other, M1, at time *t*. In this case, we draw an arrow from M1 to M2 (**Figure 1B**), which represents the Granger causality of the source node on its target. For instance, a stressed state may entail a state of being anxious or irritated. This relationship could also be reciprocal, e.g., anxiety or irritation may in turn increase stress.

The presence of two-way causal relations in this simple network with two variables may lead to a positive feedback loop in the network. In a more complex network with more nodes, this is also possible with only one-way connections, e.g., there may be a connection from stress to anxiety or irritation to stress. At a certain level of activation, these characteristic network features can theoretically promote a self-sustaining state of general high activation of several strongly interconnected variables, which could be considered a state of being locked in a certain mood, such as enduring anxiety or frustration. Such lasting negative mood states are akin to symptoms of mental disorders, which illustrates the relevance that modeling of moods and emotions may have for the understanding of psychopathology.

Each variable might also exhibit varying degrees of serial correlation, depicting how its value at time *t* + 1 is influenced by its previous values. In network representation, such a relationship is shown by looped arrows on variables indicating the autoregressive effect (**Figure 1B**).

External factors (E in **Figure 1C**) can further impact the network’s state by amplifying or dampening certain variables or even their interaction. For instance, negative events or situations, such as stressors, might have enhancing effects on negative moods or their interactions, while pleasant events/situations might dampen them, the inverse being true for positive moods. One can also imagine that social contexts, personality variables, or biological factors (e.g., drugs) can impact affective networks. We here restrict ourselves on theorizing about event- or situation-like positive or negative influences and only consider the contemporaneous effects of such external factors, i.e., the effects of E at time *t* on other variables also at time *t*. The extension of these networks to include more nodes is straightforward.

In building the model, we begin with a very minimal network containing two interacting nodes (M1 and M2) and one node for external inputs (E). This way, we avoid the complexity related to the number of parameters in the model as it grows quadratically by the number of variables in an autoregressive setting.

The evolution of states in an autoregressive model usually does not explicitly depend on time, and it is assumed that the observations are made one unit of time apart in a consecutive manner. However, EMA often violates this assumption by randomizing the observation time points within a certain time interval. It also accommodates overnight pauses, which is the only feasible way of collecting data. This latter issue leaves EMA with inherent missing data. Additionally, participants might occasionally fail to report at some time points, whether randomly or due to mood-related factors (11).

In psychological data, the use of randomized time points is generally accepted for autoregressive processes. Some researchers ignore missing points as well, aggregating data over days as a time series, and directly applying vector autoregressive (VAR) methods (12, 13). Others opt to preprocess data before applying VAR, addressing irregular time stamps, overnight gaps, and missing values through data smoothing, e.g., by interpolation (14, 15). More advanced methodologies exist to tackle these issues while handling time more effectively. Continuous time models leverage stochastic differential equations (16, 17) to model dynamics in fine-grained time scales. State-space methods project the observations into a latent space where one could adjust for time irregularities (18, 19). Similarly, dynamic structural equation models utilize a Bayesian framework to model observed and latent variable dynamics, providing techniques to handle irregular time points and missing data (20). However, these methods require more theoretical grounding than a conventional VAR system.

Aggregating daily observations into sequential time series implies a fixed network structure over time, essentially representing a trait rather than a state. While this assumption holds value for exploring individual differences, it becomes problematic when investigating within-person shifts in affect. This scenario arises when tracking the development of affective symptoms, predicting related behaviors, or assessing the impact of emotion-targeting interventions, such as emotion regulation strategies or therapy. To address this concern, time-varying VAR models have been introduced to estimate subject-specific networks that exhibit variable node connections over time. For instance, Haslbeck et al. (21) explored methods employing splines and kernel smoothing to estimate such time-varying networks. However, these approaches differ from ours in that they explicitly model time dependence, often through a combination of basic functions, whereas we implicitly characterize time-varying properties by allowing network structures to vary over days, as reflected in the random-effects variance.

Multilevel autoregressive (22–24) and VAR (25) methods have been investigated to account for the nested nature of EMA data. However, to our knowledge, their empirical performance in estimation errors, particularly for data from individual respondents, remains less explored, though they have been used on real data. Modeling individual EMA data through multilevel VAR models could also offer time-varying network structures by allowing mood interactions to exhibit random effects over days or weeks. However, this time-dependent structure is not modeled explicitly but rather in terms of the distributional characteristics of the random effects. Focused on personalized dynamic networks and through simulations, we delve deeper into the application of multilevel methods to EMA data, using an exogenous linear autoregressive mixed-effects model (LARMEx). This model extends linear mixed-effects models for normally distributed (continuous) responses, accounting for dependencies on lagged values as well as covariates within a unified framework. By employing this approach, we assume that the underlying data-generating process for EMA data follows a VAR process. This process is initiated each morning from an initial value independent of the last measurement of the previous day and evolves through a day with potentially slightly different parameters compared with other days. By this formulation, we introduce day-to-day variations in VAR coefficients (state) around a subject-specific set of values (trait), capturing this time-varying property through random effects. The subject-specific parameters constitute the model’s fixed-effects component, encapsulating the overall characteristics associated with an individual, or the trait.

This formulation can be adapted to other multilevel designs whenever there is evidence that pooling data over specific units of observations is feasible. Assuming network structures remain stable over weeks, one could consider a week as the unit of observation and aggregate daily data. Alternatively, if the network structure remains consistent across the entire respondent population, allowing individuals to exhibit slightly different parameters could involve aggregate data from each person. Here, however, we focus on single-subject analysis, assuming observations are nested in days, which in turn are nested within respondents.

## 2 Mathematical model and parameter estimation

In this section, we present the mathematical formulation of the network depicted in **Figure 1C**. We transform the model into a simple matrix form and outline the estimation procedure by formulating the maximum likelihood and restricted maximum likelihood methods. For more details, please refer to Section 2 in the **Supplementary Text**.

### 2.1 Mathematical representation

Linear autoregressive processes are used to model stochastic dynamical systems of discrete data through difference equations (26). LARMEx models the mean response as a combination of trait-like characteristics of an individual as fixed effects and state-like features varying over days (weeks) as random effects. It is important to note that existing techniques allow for the estimation of fixed effects and the variances of random effects, which in turn help predict the values of random effects. This model structure effectively accounts for the dependencies in different levels of data, such as within a day, and at the same time compensates for the lack of statistical power due to few observations on the first level (27, 28).

Let ${\text{m}}_{i,t}=\;{\left[m_{1},m_{2}\right]}_{i,t}^{T}$ be the 2 × 1 vector comprising the two mood values from the *i*th measurement day at observation occasion $t=\;1,2,\ldots ,n_{i}$ . Here, *n_i_* corresponds to the number of observations per day, and *T* denotes the transpose of a matrix. Importantly, *n_i_* might differ for every *i* despite the fact that it is normally planned to collect the same amount of data each day.

Assuming that a collection of external factors, the node E with measurements *e_i,t_*, influences mood nodes and its effect could vary over days, the network in **Figure 1B** could be modeled as a LARMEx model,


(1) $${\text{m}}_{i,t}=B_{i}^{ar}m_{i,t-1}+B_{i}^{e}e_{i,t}+B_{i}^{c}+\epsilon_{i,t}.$$

In this formulation, $B_{i}^{ar}=\beta^{ar}+b_{i}^{ar}$ are 2 × 2 matrices of auto- and cross-lagged regression coefficients; ${\text{B}}_{i}^{e}={\text{β}}^{e}+b_{i}^{e}$ could be considered as a measure of contemporaneous reactivity to external events in the long run and short periods of time, like days; and ${\text{B}}_{i}^{c}={\text{β}}^{c}+{\text{b}}_{i}^{c}$ are 2 × 1 vectors of constant terms corresponding to the two moods. Every coefficient matrix and constant term is a sum of fixed- and random-effects terms representing the subject-specific, *β*, and day-specific, *b_i_*, components. A white noise element, $\epsilon_{i,t}$ , accounts for all the uncertainties due to unknown factors, but not measurement errors. Treating the measurement errors adds another layer of complexity to the model and, hence, is avoided here for the sake of simplicity.

The random effects are assumed to be normally distributed with the mean zero and a certain variance–covariance, *b* ∼ *N*(0,*G*). Different structures of *G* are used to account for possible scenarios representing the dependencies among random effects. We restrict ourselves to a compound symmetric (CS) type where all random effects share the same variance, the diagonal elements of *G*, and the same covariance, off-diagonal terms. Furthermore, autoregressive and exogenous components are assumed to be independent. For more structures, we refer the reader to Funatogawa and Funatogawa (28) and references therein.

The selection of a diagonal form for $\epsilon_{i,t}$ primarily stems from the limited number of daily observations (29), as well as ensuring parameter identifiability. However, the total noise in the system depends also on the covariance matrix of the random effects, *G*, in Equation 4. In practice, *G* is not constrained, allowing for the potential occurrence of contemporaneous correlations between different mood variables, which is frequently observed in empirical EMA data (13).

### 2.2 Matrix form

To simplify the estimation process, we transform the autoregressive mixed-effects model into a more general format, treating lagged variables as covariates and separating fixed and random effects (30, 31). For any time point *t* within each day *i*, we have:


(2) $${\text{m}}_{i,t}=\left({\text{β}}^{ar}+{\text{b}}_{i}^{ar}\right)m_{i,t-1}+\left({\text{β}}^{e}+b_{i}^{e}\right)e_{i,t}+\left({\text{β}}^{c}+{\text{b}}_{i}^{c}\right)+\epsilon_{i,t},$$

which takes the following matrix form with separated fixed and random effects.


$$\begin{array}{c} {\left[\begin{array}{c} m_{1}\\ m_{2} \end{array}\right]}_{i,t}=\left[\begin{array}{cc} \beta_{11} & \beta_{12}\\ \beta_{21} & \beta_{22} \end{array}\right]{\left[\begin{array}{c} m_{1}\\ m_{2} \end{array}\right]}_{i,t-1}+\left[\begin{array}{c} \beta_{1}^{e}\\ \beta_{2}^{e} \end{array}\right]e_{t}+\left[\begin{array}{c} \beta_{1}^{c}\\ \beta_{2}^{c} \end{array}\right]+\\ \left[\begin{array}{cc} b_{11} & b_{12}\\ b_{21} & b_{22} \end{array}\right]{\left[\begin{array}{c} m_{1}\\ m_{2} \end{array}\right]}_{i,t-1}+{\left[\begin{array}{c} b_{1}^{e}\\ b_{2}^{e} \end{array}\right]}_{i}e_{t}+{\left[\begin{array}{c} b_{1}^{c}\\ b_{2}^{c} \end{array}\right]}_{i}+{\left[\begin{array}{c} \epsilon_{1}\\ \epsilon_{2} \end{array}\right]}_{i.t}. \end{array}$$

Considering that this equation holds true for each time point, we stack the mood values, parameters, and covariates related to the day *i* by defining:


$${\text{Y}}_{i}={\left[\begin{array}{cccccccc} m_{1,1} & m_{1,2} & \ldots & m_{1,n_{i}} & m_{2,1} & m_{2,2} & \ldots & m_{2,n_{i}} \end{array}\right]}_{i}^{T},$$

and


$$\text{β}={\left[\begin{array}{cccccccc} \beta_{11} & \beta_{12} & \beta_{21} & \beta_{22} & \beta_{1}^{e} & \beta_{2}^{e} & \beta_{1}^{c} & \beta_{2}^{c} \end{array}\right]}^{T}.$$

Consequently, by defining ${\text{b}}_{i}$ and $\epsilon_{i}$ in a similar way, the matrix representation for each day becomes


(3) $${\text{Y}}_{i}={\text{X}}_{i}\beta +{\text{Z}}_{i}{\text{b}}_{i}+\epsilon_{i}.$$

Here, *X_i_* and *Z_i_* are design matrices for fixed and random effects. In general, these matrices can have different forms depending on the included terms. In our full model, they are equal and have the following form (28):


$$X_{i}=Z_{i}={\left[\begin{array}{cccccccc} m_{1,0} & m_{2,0} & 0 & 0 & e_{1} & 0 & 1 & 0\\ m_{1,1} & m_{2,1} & 0 & 0 & e_{2} & 0 & 1 & 0\\ \vdots & \vdots & \vdots & \vdots & \vdots & \vdots & \vdots & \vdots \\ m_{1,n_{1}-1} & m_{2,n_{1}-1} & 0 & 0 & e_{n_{i}} & 0 & 1 & 0\\ 0 & 0 & m_{1,0} & m_{2,0} & 0 & e_{1} & 0 & 1\\ 0 & 0 & m_{1,1} & m_{2,1} & 0 & e_{2} & 0 & 1\\ \vdots & \vdots & \vdots & \vdots & \vdots & \vdots & \vdots & \vdots \\ 0 & 0 & m_{1,n_{1}-1} & m_{2,n_{1}-1} & 0 & e_{n_{i}} & 0 & 1 \end{array}\right]}_{i}$$

In its general form, Equation 3 for *k* moods, ***Y*** *_i_* is a *k*(*n_i_* − 1) × 1 vector of mood values; *β* is a (*k*^2 ^+ 2*k*) × 1 vector of fixed effects; ***b*** *_i_* is a (*k*^2 ^+ 2*k*) × 1 vector of random effects; and ***X*** *_i_* and ***Z*** *_i_* are *k*(*n_i_* − 1) × (*k*^2 ^+ 2*k*) design matrices. The random effects ***b*** *_i_* and residuals $\epsilon_{i}$ are assumed to be independent with a multivariate normal distribution of


$$\left[\begin{array}{c} b_{i}\\ \epsilon_{i} \end{array}\right]∼MVN\left(\left[\begin{array}{c} 0\\ 0 \end{array}\right],\left[\begin{array}{cc} \text{G} & 0\\ 0 & {\text{Σ}}_{i} \end{array}\right]\right)$$

### 2.3 Maximum likelihood

In classic linear models, the expected value of the response variable is parametrized by fixed effects. Typically, the only source of variation comes from error terms which are usually considered uncorrelated and independently distributed. This distribution gives rise to the likelihood function, and the estimation is carried out by maximizing it over the parameters. However, mixed-effects models address the correlation structure of the response variable by including random effects, which introduces further sources of variability in addition to the error terms.

Assuming the normality of both errors and random effects, mixed-effects models extend the ordinary least squares method for parameter estimation (30, 32, 33). This method has also been expanded to accommodate autoregressive terms through fixed effects (28), to account for the overall trend in data, and random effects to address further nested characteristics (22, 25).

Any inference in this context, Equation 3, involves the estimation of fixed effects, *β*, and covariance components, *G*, and **Σ***_i_*. Additionally, random effects are predicted, since *b_i_* are considered as random variables and not parameters. The underlying assumptions about the distribution of residuals and random effects impose certain restrictions on the observations, leading to the likelihood of data given the parameters and forming the basis for likelihood-based estimation methods. The observations in this formulation follow a marginal distribution given by


(4) $${\text{Y}}_{i}∼MVN\left({\text{X}}_{i}\text{β},Z_{i}\text{G}{\text{Z}}_{i}^{T}+{\text{Σ}}_{i}\right).$$

Assuming ${\text{V}}_{i}=Z_{i}\text{G}{\text{Z}}_{i}^{T}+{\text{Σ}}_{i}$ , the likelihood function for a single day reads as:


$$p(Y_{i}|\beta ,V_{i})=2\pi^{-\frac{n}{2}}|V{|}^{-\frac{1}{2}}\text{exp }\left\{-\frac{1}{2}{\left(Y_{i}-X_{i}\beta \right)}^{T}V^{-1}\left(Y_{i}-X_{i}\beta \right)\right\}.$$

In practice, data from all days are pooled, and −2 × ln *p* is used to make the estimation procedure feasible,


$$l\left(\text{β},\theta \right)=\sum_{i=1}^{N}\text{ln }|V_{i}|+\sum_{i=1}^{N}{\left(Y_{i}-X_{i}\beta \right)}^{T}V{\left(\theta \right)}^{-1}\left(Y_{i}-X_{i}\beta \right),$$

in which constants are excluded. The covariance matrix is typically parameterized and factorized as ${\text{V}}_{i}={\text{V}}_{i}\left(\theta \right)=L^{T}L$ , using an upper triangular matrix *L* to ensure that the estimated covariance is a positive definite matrix, overcoming computational difficulties associated with determinants and inverses.

If the covariance is known, the generalized least square estimator of β that minimizes l(β,θ) is given by


$$\hat{\beta }={\left[\sum_{i=1}^{N}{\text{X}}_{i}^{T}V_{i}^{-1}{\text{X}}_{i}\right]}^{-1}\sum_{i=1}^{N}{\text{X}}_{i}^{T}{\text{V}}_{i}^{-1}\beta_{i},$$

in which a generalized inverse is used if the sum is not invertible. This estimator is asymptotically unbiased, i.e., $E(\hat{\beta })\;=\beta$ , and follows a multivariate normal distribution if the response variable has a conditional normal distribution (27), with


$$Cov\left(\hat{\beta }\right)={\left[\sum_{i=1}^{N}X_{i}^{T}V_{i}^{-1}X_{i}\right]}^{-1}.$$

The estimation of *β* is substituted in the likelihood function, and the result is maximized with respect to *V_i_* (*θ*) to derive the covariance estimator. This procedure typically employs iterative methods such as Newton–Raphson or expectation–maximization algorithms (34). It is well known that the maximum likelihood estimators can exhibit biases in finite samples. Therefore, restricted maximum likelihood is used to correct for the loss of degrees of freedom resulting from the estimation of *β*.

Upon estimating these parameters, the prediction of random effects follows from their conditional mean given the responses *Y_i_*,


$$E(b_{i}|Y_{i})=GZ_{i}^{T}V_{i}^{-1}\left(Y_{i}-X_{i}\hat{\beta }\right),$$

This gives rise to the so-called best linear unbiased predictor (BLUP) (33, 35),


(5) $${\hat{\text{b}}}_{i}=\hat{\text{G}}{\text{Z}}_{i}^{T}{\hat{\text{V}}}_{i}^{-1}\left({\text{Y}}_{i}-{\text{X}}_{i}\hat{\beta }\right).$$

## 3 Simulations

In this section, we utilize simulations to explore the effectiveness and feasibility of our approach by assuming that the underlying true data-generating process is the mixed-effects system outlined in Equation 1. We generate synthetic data by setting predetermined fixed effects and the variance of random effects. By varying noise intensities, the number of observation days, and compliance rates, we cover a range of scenarios commonly encountered in real-world datasets. The known parameters are called “true” values, while fitting LARMEx to these datasets using the Julia (36) package *MixedModels.jl* (37) provides the “estimated” parameters. *MixedModels.jl* mirrors the functionality of the *lme4* package in R, harnessing the substantial speed advantages afforded by the Julia programming language. In each instance, our simulations involve generating realizations from a mood network consisting of two moods and an external factor node. The primary objective is to assess how well the true parameters are recovered in this context.

### 3.1 Data-generating process

To establish a foundation for our simulations, we define the fixed effects, including the matrix of autoregressive coefficients, the vector of coefficients for exogenous variables, and constant terms in Equation 2 as follows. For a more comprehensive understanding, kindly refer to the **Supplemental Material** for detailed information.


(6) $$\beta^{ar}=\left[\begin{array}{cc} 0.3 & -0.3\\ -0.3 & 0.3 \end{array}\right],\;\beta^{e}=\left[\begin{array}{c} 0.3\\ -0.3 \end{array}\right],\;\beta^{c}=\left[\begin{array}{c} 0\\ 0 \end{array}\right].$$

This set of parameters defines a moderately connected mood network of a hypothetical person, assumed to be of a trait-like nature that is preserved over long periods, like weeks or months. The effects of the exogenous factors, *β^e^*, are set at the same order of magnitude as the autoregressive coefficients. To maintain the trajectories mostly within the range of [−1,1], the initial values and the intensity of external factors are uniformly chosen from [−0.5,0.5] and [0,0.5], respectively. Random effects are chosen to be independent, following a normal distribution with a mean zero.

**Figure 2:**
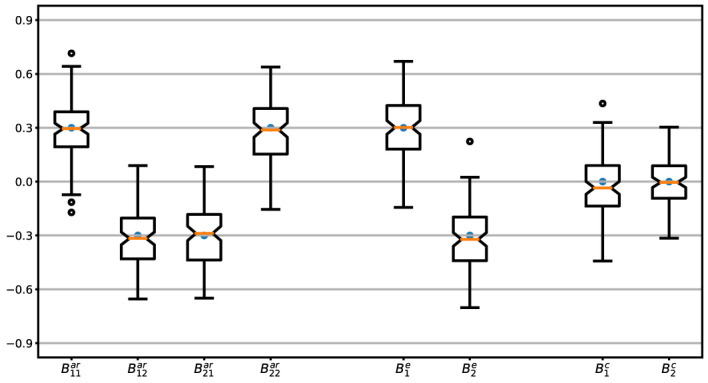
Parameter values. Distributions of the parameters, $B_{\cdots }^{\cdots }=\beta^{\cdot \cdot \cdot }+b_{\cdots }^{\cdots }$ , and sum of fixed and random effects for 200 simulated days. Each boxplot shows the interquartile range (IQR) notched over the median and whiskers extending 1.5 × IQR beyond the first and third quartiles. Fixed effects are shown by dark points.

To ensure the stability of the autoregressive processes, the elements of $\beta^{ar}+b_{i}^{ar}$ must satisfy the criterion that all the eigenvalues are smaller than one. This is achieved by generating a large sample compared with the number of days, 20,000 in this case, and then retaining all sets for which the eigenvalues fulfill the stability condition. However, this step can become challenging, as it might lead to deviations from a normal distribution in the resulting random effects. This becomes even more demanding when we impose theoretical constraints on the network structure, such as positive self-loops and negative values for edges between positive and negative moods, and leaves a narrow range for random effects ending up in low variances. Therefore, we relax the latter requirement and allow some edges in the networks to take values of the opposite sign than seen in **Figure 1C**. The distributions of the sum of fixed and random effects are shown in **Figure 2**, for which the covariance of random effects at the population level is an 8 × 8 diagonal matrix, *G* = 0.03 × *I*_8×8_, where *I* denotes the identity matrix.

With these considerations, in 78% of the cases, the structure of the network is preserved as **Figure 1C**, i.e., with the same sign of edges as illustrated by different colors. The noise is assumed to be of the form *σ*^2^*I* where we run the simulations for low, moderate, and high noise intensities characterized by the variance of noise and the signal-to-noise ratio (SNR) as (*σ*^2^*,SNR*) ∈ {(0.01,12),(0.02,6),(0.06,2)}. These values are valid for the chosen *G* which remains consistent throughout our analysis. It is important to note that higher SNR values can be achieved by increasing the variance of random effects. In each case, we calculate SNR as the ratio of the variance of the noiseless signal to that of the noise (38). That is, for Equation 1,


$$SNR=\frac{Var\left({\text{B}}_{i}^{ar}{\text{m}}_{i}+{\text{B}}_{i}^{e}e_{i}+{\text{B}}_{i}^{c}\right)}{\sigma^{2}}.$$

We refrain from transforming the SNR into decibel (dB) units, common in signal processing, because of its unfamiliarity in the field. More details are provided in Section 4, **Supplementary Text**.

This work is part of a study involving the collection of EMA data from 250 subjects, 10 observations per day over the first weeks (6 days) of six consecutive months (39). Therefore, we generate data while varying the number of days, *N*, from the set {4, 6*,…*, 36} to assess the impact of additional EMA days on parameter estimation. The mean number of longitudinal observations per day, $\bar{n}=\sum_{i=1}^{N}n_{i}/N$ has a lower bound to ensure identifiable random effects. Given a network of *k* moods and one node of external factors, one has to predict a total number of (*k*^2 ^+ 2*k*)*N* random effects while there are $k\sum_{i=1}^{N}(n_{i}-1)$ observations. A rule-of-thumb identifiability criterion for mixed-effects models is that the number of observations must surpass the number of random effects (33). Consequently, the number of moods in the aforementioned network should be less than $\bar{n}\;-\;2$ . Therefore, for an empirical EMA study with at most 10 observations per day and a presumed compliance rate of 70%, this method would be suitable for small mood networks up to four or five nodes.

### 3.2 Parameter recovery

To assess the effectiveness of parameter recovery, we employ the LARMEx model on datasets generated with varying combinations of the previously mentioned parameters.

**Figure 3:**
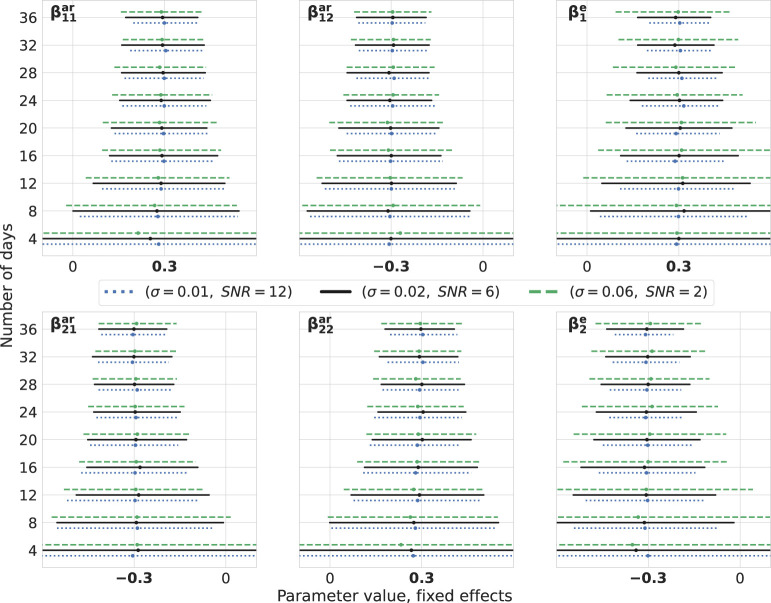
Fixed effects: bootstrap. Estimations of fixed effects for different levels of noise intensity and number of days. The number of observations per day is 10. True values of parameters are highlighted in bold on the *x*-axes. For every number of days on the vertical axes, three lines are drawn representing the bootstrap 95% confidence intervals around the median depicted by cross signs. Every line is color- and style-coded to demonstrate one noise intensity. Data are generated for one simulated subject, 1,000 times repeatedly, with (*σ*^2^, SNR) ∈ {(0.01,12),(0.02,6),(0.06,2)}, and estimations are performed using the *MixedModels.jl* package in Julia.

**Figure 4:**
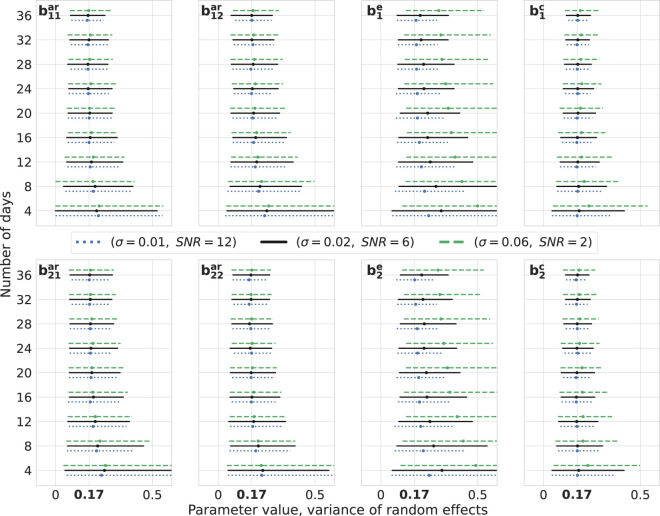
Variance of random effects: bootstrap. Estimations of the variance of random effects for different levels of noise intensity and number of days. The number of observations per day is 10. True values of parameters are highlighted in bold on the *x*-axes, $\sqrt{0.03}\;\approx \;0.17$ . For every number of days on the vertical axes, three lines are drawn representing the bootstrap 95% confidence intervals around the median depicted by cross signs. Every line is color- and style-coded to demonstrate one noise intensity. Data are generated for one simulated subject, 1,000 times repeatedly, with (*σ*^2^, SNR) ∈ {(0.01,12),(0.02,6),(0.06,2)}, and estimations are performed using the *MixedModels.jl* package in Julia.

We construct bootstrap confidence intervals, which are used to quantify the sampling distribution of the estimates. For the fixed effects detailed in Equation 6, we generate random effects for 200 simulated days. Subsequently, for any given *n* number of days, parameters are sampled from this set. Throughout each iteration, we create independent datasets for the three noise intensities mentioned earlier, repeating this process 1,000 times. The parameter estimation is carried out using the *MixedModels.jl* package in Julia. The resulting confidence intervals for both fixed effects (**Figure 3**) and variances of random effects (**Figure 4**) are presented. For clarity, we display the results for the number of days from the set 4, 8*,…*, 36.

Upon visual inspection, it becomes evident that by adding more days of observations, the confidence intervals become narrower and their widths decrease for lower noise intensities. It is worth noting that, for these levels of SNR, they do not differ drastically. It is also noteworthy that parameters corresponding to the exogenous variable are less precise compared with others. Furthermore, this methodology can be harnessed for conducting power analysis by setting an appropriate null hypothesis, e.g., in terms of a cutoff for a certain parameter. Then, the percent of the truly rejected null hypothesis, when it is indeed false, would give an approximate power (40, 41).

**Figure 5:**
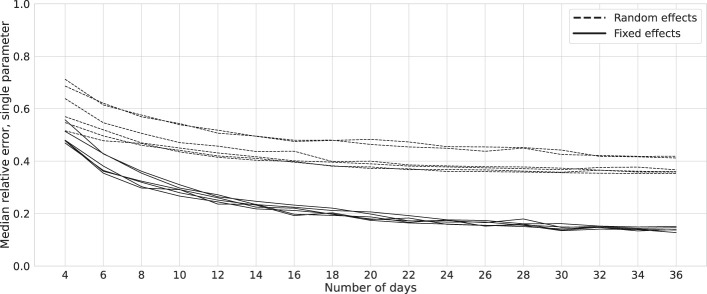
Relative errors: bootstrap. Solid and dashed lines represent the mean relative estimation errors for the six parameters of a network as in **Figure 1C**. Data are generated for one simulated subject, 1,000 times repeatedly, with (*σ*^2^, SNR) = (0.02, 6), and estimations are performed using the *MixedModels.jl* package in Julia. The two lines with higher relative errors correspond to the exogenous factors.

We compute the relative estimation error (REE) in the aforementioned cases as $\delta_{\theta }=\;|\hat{\theta }-\theta \left|/\right|\theta |$ in which *θ* and $\hat{\theta }$ represent the true and estimated parameters, respectively. To avoid computational difficulties, only random effects larger than 0.02 are considered in these analyses. We also report only the coefficients present in a network representation, i.e., *β^ar^*, *β^e^*, *b^ar^*, and *b^e^*. This analysis underscores that in a mixed-effects model the prediction of random effects is not as reliable as the estimation of fixed effects in terms of being able to recover the parameters varying over days in our case. The reason is that only the variance of random effects is present in the likelihood function and individual values are not estimated directly. Notably, software outputs such as those from packages like *MixedModels.jl* in Julia or *lme4* in R hold only for the expected values, Equation 5, and should not be considered as singular parameter estimations. **Figure 5** illustrates that the median REE for random effects approaches approximately 40% as the number of days increases, with minimal change after approximately 20 days. Conversely, the error for fixed effects remains below 20%, implying that with a reasonable dataset, improved fixed-effects estimates can be anticipated. Nonetheless, this outcome remains suboptimal. Notably, the two dashed lines in **Figure 5** represent exogenous factor coefficients, which exhibit comparably poorer estimates than others (refer to **Figures 3**, **4**).

**Figure 6:**
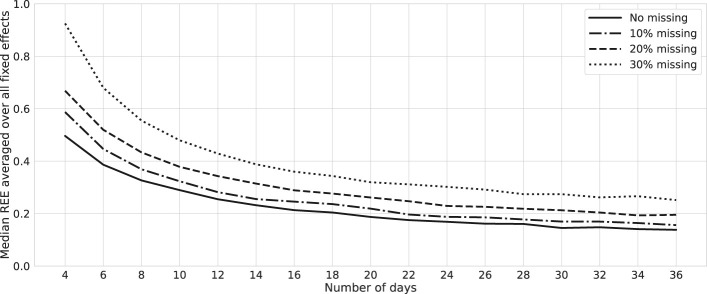
Relative errors: bootstrap with missing values. Lines represent the median relative estimation errors (REEs) averaged over fixed-effect parameters of a network with two mood nodes and one node of external factors in the presence of missing values at random. Data are generated for one simulated subject, 1,000 times repeatedly, with (*σ*^2^, SNR) = (0.02, 6). We remove 10%, 20%, and 30% of the observations randomly for each case, and estimations are performed using the *MixedModels.jl* package in Julia.

Missing values are a common occurrence in EMA data. For our study, we consider the presence of randomly missing values, leading to the omission of 10%, 20%, and 30% of observations. Employing the same methodology as earlier, we replicate the analysis for a two-variable mood network. We calculate REEs for varying numbers of days and the three distinct levels of missing values. The collective average of these REE values is succinctly presented in **Figure 6**. This insight reveals a potentially non-linear influence of missing data, which may be attributed to the practice of listwise deletion, in conjunction with the restriction to consecutively recorded data points. As compliance rates decline, the probability of consecutively recorded data instances experiences a significant decrease. This underscores the necessity of considering imputation techniques depending on the severity of missing values.

**Figure 7:**
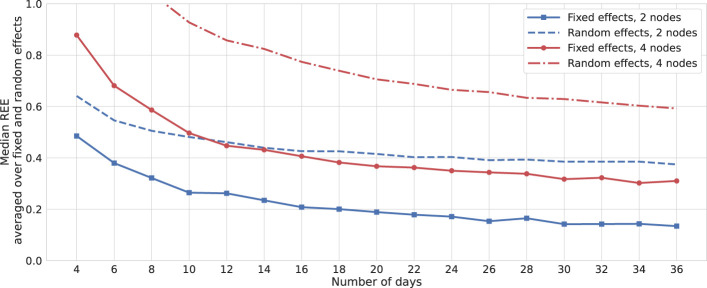
Relative errors: bootstrap. Solid and dashed lines represent the median relative estimation errors (REEs) averaged over fixed- and random-effect parameters, respectively. The results are depicted with similar colors for networks with two and four mood nodes and one node of external factors. Data are generated for one simulated subject, 1,000 times repeatedly, with SNR = 6 for both cases, and estimations are performed using the *MixedModels.jl* package in Julia.

To assess the scalability of this method in higher dimensions, we simulate a network comprising four interacting nodes—two positive and two negative valences—along with an external factor influencing all mood variables. **Figure 7** presents the REEs for this simulation and compares them to those in **Figure 5**, averaging REEs across all fixed and random effects. Notably, for a two-fold increase in the number of mood variables, the REEs for fixed effects almost doubled. This observation suggests that, for smaller networks, employing the full model might be more appropriate. However, for larger networks, it would be advisable to opt for reduced models containing a smaller number of random effects. Additionally, dimension reduction techniques could be applied to merge nodes that measure similar psychological constructs.

## 4 Conclusion

We studied an extension of linear mixed-effects models by adding an autoregressive component to model the network representation of affective state which is suitable to model intensive longitudinal data, the so-called EMA acquired by experience sampling methods. Specifically, we assumed the simplest possible network of two causally interacting state nodes under the influence of external factors represented by a node acting on both states in a contemporaneous fashion. We detailed the mathematical formulation of this representation and discussed its components by generating data in a step-by-step manner. Subsequently, we transformed it to a classic linear mixed-effects form and summarized the foundations of parameter estimation by likelihood function.

This extension has been previously introduced as a multilevel model with daily observations nested in days, days nested in respondents, and respondents nested in a sample of subjects (24, 25). Given that our objective was constructing individualized networks, we employed a two-level model with daily observations nested in days for a specific respondent and estimated subject-specific mood networks that may possess a time-varying structure depending on the variance of random effects. Using simulated data, we constructed bootstrap confidence intervals for the fixed-effect parameters, offering insight into the behavior of the model as more days are added to the observation. We also demonstrated how estimation precision is affected when dealing with a missing completely at random scenario as compliance rates decrease. In this analysis, missing values were addressed through listwise deletion. We argued that random effects are not directly estimated within this framework and highlighted the difference between the relative estimation errors for fixed and random effects. This difference revealed that, within a mixed-effects model, random effects are less precisely identified, with marginal gains in precision as the number of observations increases. Therefore, in order to infer individual networks from EMA, one should build a two-level model with daily observations nested in days for a single respondent. Our results, indicating approximately double relative estimation errors for a network with four nodes compared with one with two nodes, suggest cautious application of this approach in higher dimensions.

While benefiting from the capabilities of mixed effects and autoregressive models, it is crucial to acknowledge a notable limitation of this approach. Linear autoregressive models are unable to reveal complex characteristics of a dynamical system, such as bistable points, crucial to studying transitions between different stable states, like longer-term periods of fixed moods or shifts between states of health and mental disorder (42, 43). Moreover, we presumed that networks have time-varying characteristics, but unlike Haslbeck et al. (21), the time dependence was not formulated explicitly, opting instead to capture it indirectly via the variance of random effects. Nonetheless, it is reasonable to assume that the interactions or the impact of external forces changes over time in a specific way. A simple time dependency might be achieved by adding slopes to the model which capture the linear time trend of the corresponding parameter, ${\left[\beta_{\ldots }+b_{\ldots }\right]}_{slo}$ . Another more sophisticated approach could involve letting parameters follow a random walk, $\theta_{t}=\theta_{t}-1+\eta_{t}$ , or making the variance–covariance structures time-dependent, $\sum_{t}=F_{t}D_{t}{F^{\prime }}_{t}$ . The Markov chain Monte Carlo or state-space techniques are usually used for estimating such models (44, 45).

In a more realistic setting, one could also assume that external forces act not only on states but also on their interactions in an autoregressive way. This assumption results in having arrows pointing to some edges in the network representation. An additional equation must then be added to Equation 1, leading to a non-linear system,


$$B_{i,t}^{ar}=\alpha_{i}^{ar}B_{i,t-1}^{ar}+\alpha_{i}^{e}e_{i,t}+\alpha_{i}^{c}+\eta_{i,t},$$

in which *η_i_* are assumed to be white noises and mutually uncorrelated at all leads and lags to other sources of variability in the model. This formulation implies time-varying autoregressive coefficients. However, these methods as discussed in the literature require long time series to achieve acceptable results, and the feasibility of mixed-effects modeling requires further exploration.

In a broader context, it would be valuable to extend this research to encompass more comprehensive real-world scenarios and investigate the model’s performance under various conditions. This could involve exploring the impact of different network structures, the presence of external influences on interactions between mood nodes, and the incorporation of more complex temporal dependencies. Additionally, the application of this approach to actual EMA datasets would provide insights into the method’s practicality and effectiveness in real-world contexts.

We primarily considered one aspect of study design relating to sample size, varying the number of observations per subject by adding more days. We also showed how sensitive such a model might be to the intensity of noise in data. In summary, while autoregressive mixed-effects models offer a representation for affective networks, estimating individualized parameters remains a challenge within common longitudinal designs, where obtaining the required number of observations per individual for acceptable accuracy might be unattainable.

## Data availability statement

The original contributions presented in the study are included in the article/supplementary material. Further inquiries can be directed to the corresponding author. The authors offer a comprehensive outline of the data-generation process implemented in Julia programming language as a supplementary text, and supply tools for conducting simulations and fitting empirical data. Recognizing the widespread use of R within the field, the authors also provide a graphical user interface in R’s Shiny platform as a toolbox targeting end users working on empirical data with limited or no programming knowledge. This toolbox comprises source codes and a web application hosted on a server. The associated source codes and detailed manuals are conveniently stored within two distinct public Git repositories, accessible through the following provided links: Julia source code: https://github.com/spooseh/MixedEffectsVAR; R’s Shiny source code: https://github.com/spooseh/larmexShiny; Web app: https://spooseh.shinyapps.io/larmexShiny/.

## Author contributions

All authors contributed to the study conceptualization. SP drafted the manuscript and performed the analyses. RK substantially contributed to the drafting. All authors contributed to the article and approved the submitted version.
