# Supplementary material for: Intraindividual time-varying dynamic network of affects: linear autoregressive mixed-effects models for ecological momentary assessment
Source: Front Psychiatry. 2024 Mar 20;15:1213863. doi: 10.3389/fpsyt.2024.1213863 (PMC10997345; doi:10.3389/fpsyt.2024.1213863)
Supplement: Supplementary file 1 [file DataSheet_1.pdf]

Supplementary Material

Summary

This document outlines the development of an external linear autoregressive mixed-effects model, designed for estimating dynamic networks of personal emotions over time. This model is suitable for data collected through ecological momentary assessment.

We begin by explaining the theoretical foundation, followed by a detailed presentation of the construction of data types and functions. These components represent the process of generating the data and the step-by-step approach to fitting the model. For fitting mixed-effects models, we employ *MixedModels.jl* in *Julia*, and *lme4* in *R* programming languages.

Considering that our study is entirely based on simulations and recognizing *Julia*'s enhanced computational capabilities, we have implemented the tools and conducted the analysis using the *Julia* language. However, acknowledging the widespread usage of *R* in the field, we have also developed a user-friendly graphical interface. This interface leverages the *Shiny* platform within *R*, providing an accessible means of fitting real data without requiring programming skills. This interface is hosted on a server, serving as a toolbox tailored for users with limited programming experience.

For convenience, we have made the source codes for both implementations available in separate GitHub repositories. The following links provide access to them and the web application.

- Julia: github.com/spooseh/MixedEffectsVAR
- Shiny, source code: github.com/spooseh/larmexShiny
- Shiny web app: spooseh.shinyapps.io/larmexShiny/

1 INTRODUCTION

We consider simple directed networks comprising two moods and one external factors node. The two moods interact mutually by enhancing (blue) or dampening (red) each other under the unidirectional influence of external factors.

Assuming intensive longitudinal data through Ecological Momentary Assessment (EMA), we formalize the mathematical representation of these networks, Figure S1, by an Exogenous Linear Autoregressive Mixed-effects model (LARMEx). The autoregressive coefficients represent the interactions and the external factors are treated as exogenous covariates. We let every parameter in the model have fixed and random components aiming at networks that are allowed to have variable structures over reasonable units of

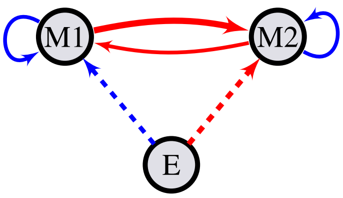

Figure S1: Nodes $M1$ and $M2$ interact with each other leading to temporal correlation, meaning that the value of the source node (variable) at time t has an impact on the value of its target at time $t+1$. Looped arrows indicate autoregressive effects. Exogenous factors influence moods at the same time in a one-way fashion. Blue and red arrows indicate activation and suppression respectively. Different thicknesses stand for distinct interaction strengths. Dashed arrows indicate contemporaneous effects while solid ones are indicative of possible temporal causalities.

time like days or weeks depending on the study design. Harnessing the rich theoretical and computational literature that furnishes classic linear mixed-effects models, we transform this formulation to a classical one and use the existing methods and tools. We assume our model is the true data generating process and simulate data using a predefined set of parameters. Then we investigate the performance and feasibility of this approach in delivering reliable estimates for different choices of the number of observations and the intensity of noise.

2 MATHEMATICAL REPRESENTATION

Linear autoregressive (AR) processes are used to model stochastic dynamical systems of discrete data through difference equations. That is, the current state of the system, s_t, is assumed to depend linearly on the preceding states, $s_{t-1}, \ldots, s_{t-p}$ and a constant term, β_0, analogous to the intercept in linear regression. All the unknown factors which might affect the system are encapsulated in the error term ϵ_t and ϵ_t is usually assumed to be white noise with zero mean (Hamilton, 1994),

$$s_t = \sum_{l=1}^{p} \beta_l s_{t-l} + \beta_0 + \epsilon_t. \tag{S1}$$

In this formulation, s is usually supposed to be a time series comprising sufficient observations over time, which makes it possible to detect its temporal dynamics. The autoregressive coefficients β_i determine how the current value of s is affected by its preceding values by p steps back in time. In the simplest form of an AR process, the dependence on previous states is only taken one step back and the so-called *Lag(1)* processes are formulated by $s_t = \beta_1 s_{t-1} + \beta_0 + \epsilon_t$. In the absence of noise, this process has a fixed point of $\frac{\beta_0}{1-\beta_1}$. If such a process reaches its fixed point it will stay there forever. Otherwise, such a point determines the asymptotic behavior of the process in the long run. In this work we only consider stable systems that will eventually converge to fixed points and require that $|\beta_1| < 1$ for a single variable *Lag(1)* process.

The immediate generalization of AR processes to accommodate multiple states is a common practice whenever one needs to study the dynamics of several variables simultaneously. Exogenous covariates might also be included in a linear fashion to account for the effects of known external factors.

LARMEx models the mean response as a combination of trait-like characteristics of an individual as fixed effects (FE) and state-like features varying over days (weeks) as random effects (RE). Needless to say, existing techniques allow for the estimation of fixed effects and the variances of random effects, and the values of random effects are predicted using these estimations. This way, the model accounts for the dependency in different levels of data, e.g. during a day, and at the same time compensates for the lack of statistical power due to few observations on the first level (Funatogawa and Funatogawa, 2018; Fitzmaurice et al., 2011).

We build a dynamic affect network comprising two interacting nodes (e.g., positive and negative mood in simplification) and an exogenous factor (E) representing an emotionally meaningful event or situation that may be considered as the input for the dynamical system.

2.1 Mood Networks

Let $\boldsymbol{m}_{i,t} = [m_1, m_2]^T_{i,t}$ be the 2×1 vector corresponding to the two mood values from the ith measurement day at any observation occasion $t = 1, 2, \ldots, n_i$ where n_i is the number of observations per day and T denotes the transpose of a matrix. Note that n_i might differ for every i despite the fact that it is planned to collect the same amount of data every day. In the absence of external driving forces like pleasant or unpleasant events and noise, the mood network (Figure S2a) is represented by a first-order autoregressive system

$$\boldsymbol{m}_{i,t} = \boldsymbol{B}^{ar}_i \boldsymbol{m}_{i,t-1} + \boldsymbol{B}^c_i, \tag{S2}$$

in which $\boldsymbol{B}^{ar}_i = \boldsymbol{\beta}^{ar} + \boldsymbol{b}^{ar}_i$ are 2×2 matrices of auto- and cross-lagged regression coefficients, and $\boldsymbol{B}^c_i = \boldsymbol{\beta}^c + \boldsymbol{b}^c_i$ are 2×1 vectors of constant terms corresponding to the two moods. Every coefficient matrix and constant term is a sum of fixed- and random-effects terms representing the subject specific, $\boldsymbol{\beta}$, and day specific, $\boldsymbol{b}_i$, components. The random effects are assumed to be normally distributed with the mean zero and a certain variance-covariance, $\boldsymbol{b} \sim N(0, \boldsymbol{G})$. Different structures of $\boldsymbol{G}$ are used to account for possible scenarios representing the dependencies amongst random effects. We restrict ourselves to a compound symmetric (CS) type where all random effects share the same variance, the diagonal elements of $\boldsymbol{G}$, and the same covariance, off-diagonal terms. Furthermore, autoregressive and exogenous components are assumed to be independent. For more structures we refer the reader to (Funatogawa and Funatogawa, 2018) and references therein.

To avoid the complexity of restricting moods to take only positive values, we assume that they are centered and can take any value. Nevertheless, we set the initial values, constant terms and the range of exogenous factors in a way that for reasonably low noise

levels, most of the trajectories remain in the interval $[-1, 1]$. The dynamics represented by Equation S2 is asymptotically stable given that all the eigenvalues of $\boldsymbol{B}_i^{ar}$ have modulus less than one, Figure S2b (Lütkepohl, 2005). The asymptotic behavior is determined by the autoregressive coefficients and constant terms. Note that the coefficients take positive and negative values so the moods interact through activating and suppressing each other, although in a more complex networks there might be only positive or negative interactions. We also assume that the constant term has a zero fixed-effect component to reduce the number of parameters. In dealing with real data we put no constraint on the coefficients to account for any possibility.

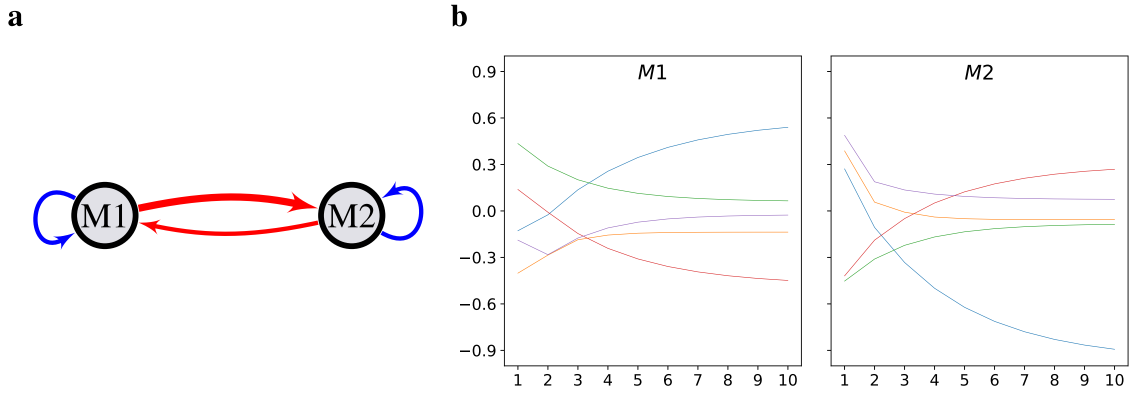

Figure S2: Tow interacting nodes. Network of two nodes M1 and M2, e.g. positive and negative mood, without external forces (a), realizations for 5 simulated days of 10 observation each (b). Data is generated using Equation S2 with initial values for different days sampled uniformly from the interval $[-0.5, 0.5]$.

In Figure S2b, depending on the magnitudes of coefficients, different patterns of decay are seen. For larger values, variables take a longer time to relax toward the asymptotic level.

2.2 Network with Exogenous Factors

Assuming that a collection of external factors, E, acts on mood nodes and its effect could vary over days, the network of moods as discussed above could be extended in two ways (Figure S3a). On the one hand, we could assume that on every day a subject is exposed to a baseline level of input, e.g. stressors, which might be different for every day. This has already been treated by adding a constant term, $\boldsymbol{B}_i^c = \boldsymbol{\beta}^c + \boldsymbol{b}_i^c$ as fixed and random intercepts for day i, Equation S2. On the other hand, one could assume that E has a time-dependent component as well, which is measured along with the moods. For instance, a day could be marked by various unpleasant events or alternating positive and negative events. This term enters as a time-varying covariate to the formulation in Equation S2 and forms an exogenous autoregressive process of

$$\boldsymbol{m}_{i,t} = \boldsymbol{B}_i^{ar}\boldsymbol{m}_{i,t-1} + \boldsymbol{B}_i^e\boldsymbol{e}_{i,t} + \boldsymbol{B}_i^c. \tag{S3}$$

In this formulation, $\boldsymbol{B}_i^e$ could be considered as a measure of contemporaneous reactivity to external events in the long run and short periods of time, like days. The term $\boldsymbol{B}_i^e \boldsymbol{e}_{i,t} + \boldsymbol{B}_i^c$ determines how the asymptotic values of moods are affected by external amplifiers. Since E varies over time, the asymptotic levels are changing from time to time and, therefore, a fluctuating pattern emerges over the mood trajectories. In this way, the differences in exposure to stressors, for instance, are accounted for. The stressor is measured longitudinally alongside moods. In Figure S3b below, we included E as an impulse, i.e., it has a nonzero value at $t = 5$ and is zero elsewhere. This is done only for presentation purposes, and in practice, it might take any value at different points. Opposite perturbations in the mood levels are visible compared to Figure S2b.

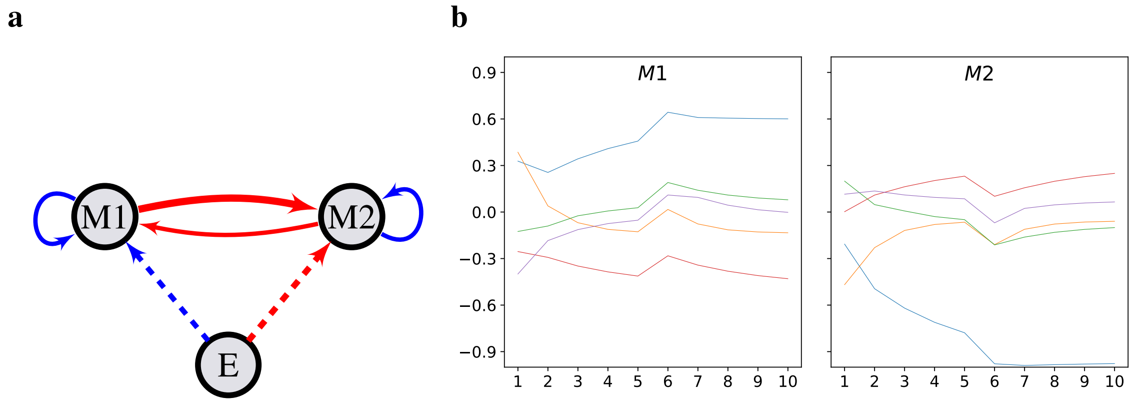

Figure S3: Tow interacting nodes with external factors. Network of two nodes M1 and M2, e.g. positive and negative mood, with a fixed amount of negative external input E acting on both at time point 5 (a), realizations for 5 simulated days of 10 observation each (b). An analogous model could be postulated for positive events or situations as inputs.

In a more realistic scenario, one would introduce a positive random value at each time point with a non-negative value as the fixed effect and generate random effects from a truncated normal distribution to keep the elements of $\boldsymbol{e}_{i,t}$ positive. If this was the case an overall upward (downward) profile would be visible in data indicating the exciting (inhibiting) effect of the stressors on negative (positive) mood.

2.3 Unknown Effects as Noise

So far we considered only deterministic models where the dynamics of the system is fully known once the parameters are fixed. However, our knowledge of real systems is usually imperfect either under the influence of measurement errors or lack of information about unknown influences on the dynamics. To account for all the uncertainties due to unknown factors, but not measurement errors, we add a white noise element, $\boldsymbol{\epsilon}_{i,t}$, to Equation S3. Treating measurement errors adds another layer of complexity to the model and hence is avoided here for the sake of simplicity. Then the full model of mood networks in the presence of external factors as an exogenous linear autoregressive mixed-effects model

becomes

$$\boldsymbol{m}_{i,t} = \boldsymbol{B}_i^{ar}\boldsymbol{m}_{i,t-1} + \boldsymbol{B}_i^{e}\boldsymbol{e}_{i,t} + \boldsymbol{B}_i^{c} + \boldsymbol{\epsilon}_{i,t}, \tag{S4}$$

in which $\boldsymbol{\epsilon}_{i,t} \sim N(\boldsymbol{0}, \sigma^2\boldsymbol{I}_{2\times 2})$. The choice of a diagonal form for $\boldsymbol{\epsilon}_{i,t}$ guarantees the model parameters be identifiable. However, the total noise in the system depends also on the covariance matrix of the random effects, $\boldsymbol{G}$ in Equation ??. Given that in practice there is no restriction on $\boldsymbol{G}$, any potential contemporaneous correlation between different mood variables, as empirical EMA data usually exhibit (Epskamp, 2020), could occur as well. Taken E into account, there might be an increasing variance and divergent profiles up- and downwards. Adding this noise to the system results in Figure S4.

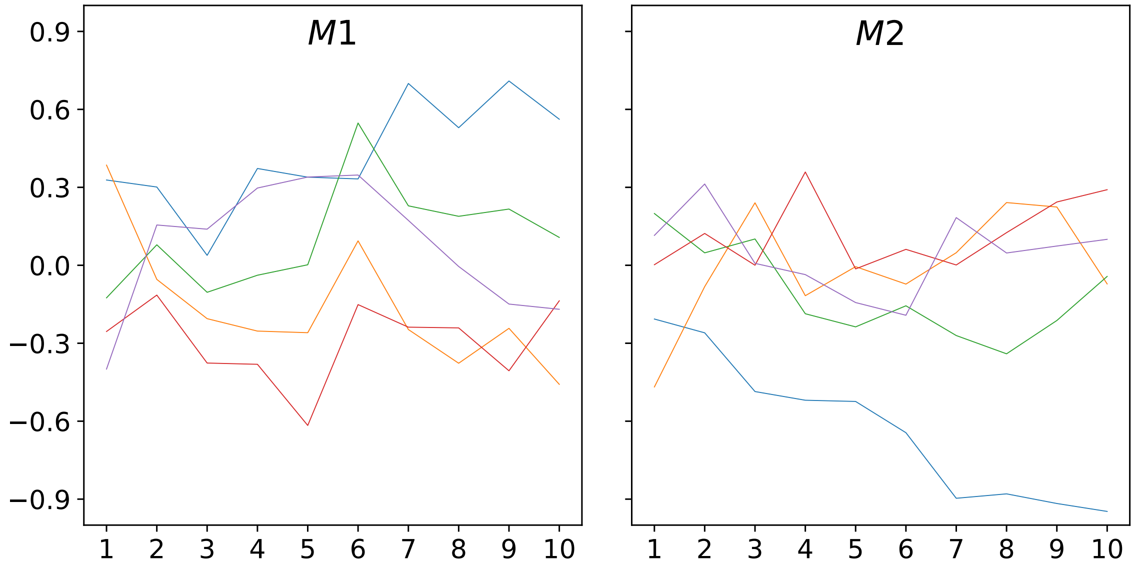

Figure S4: Tow interacting nodes with external factors and noise. Realizations from a network of two nodes M1 and M2, e.g. positive and negative mood, for 5 simulated days of 10 observation each. External factors act on both nodes at time point 5 in the presence of noise.

3 DATA GENERATING PROCESS

Let $m_{i,t} = [m_1, m_2]_{i,t}^T$ be the 2×1 vector corresponding to the mood values from the ith day at any observation occasion $t = 1, 2, \ldots, n_i$ where n_i is the number of observations per day and T denotes the transpose of a matrix.

Assuming that a collection of external factors (E) act on mood nodes and their effect could vary between days, the evolution of $m_{i,t}$ is represented by a LARMEx model as

$$m_{i,t} = (\beta^{ar} + b_i^{ar})m_{i,t-1} + (\beta^{e} + b_i^{e})e_{i,t} + (\beta^{c} + b_i^{c}) + \epsilon_{i,t},$$

in which β and b represent the fixed and random effects (FE and RE henceforth). The temporal (Granger-causal) connections between the moods are governed by the lag(1) autoregressive term, $(\beta^{ar} + b_i^{ar})m_{i,t-1}$. The remaining terms represent the contemporaneous

effects of E and the constants (intercepts). For simplicity, we assume no connections from moods to E.

4 GENERATING A SET OF KNOWN PARAMETERS

For simulating data with this model, we need to specify the parameters. To achieve replicability, one can predefine a random number generator (RNG). In Julia, we implement this using the following `struct`.

```
mutable struct parLARMEx
    nAR::Int                  # number of temporally connected nodes
    rng::AbstractRNG          # one RNG for consintency
    nL2Max::Int               # how many RE to generate
    nSamp::Int                # sample size for generating REs
    B_AR::Matrix{Float64} # FE autoregressive coefficients
    B_E::Vector{Float64}  # FE coefficients of exogenous factors
    b_var::Matrix{Float64}# variance of group of RE
    b_cov::Matrix{Float64}# variance-covariance matrix of RE
    b_ar::Matrix{Float64} # RE for autoregressive coefficients
    b_e::Matrix{Float64}  # RE for exogenous coefficients
    b_c::Matrix{Float64}  # RE for constant terms
end
```

To generate instances of this `struct`, a constructor is implemented as

```
parLARMEx(;b=[], nAR=2, rng=MersenneTwister(), nL2Max=100,
           nSamp=20000, B_ar=.3, B_e=.3,  b_var=[.03 .03 .03])
```

where the keyword arguments are:

- `b = []`: if provided with a matrix, RE are extracted from it otherwise generated
- `nAR = 2`: number of temporally connected nodes
- `rng = MersenneTwister()`: if left out, a `MersenneTwister` RNG is produced anew, otherwise a custom one should be provide for consistency and replication
- `nL2Max = 100`: how many RE to generate
- `nSamp = 20000`: initial sample size for generating RE, see genRE_CS() for explanation
- `B_ar = .3`: absolute value of FE autoregressive coefficients, `= []` for random values between 0.1 and 0.6
- `B_e = .3`: absolute value of FE exogenous coefficients, `= []` for random values between 0.1 and 0.6
- `b_var = [.03 .03 .03]`: for constructing a default variance-covariance of RE

For simplicity we restrict the fixed effects to be

$$\beta^{ar} = \begin{bmatrix} 0.3 & -0.3 \\ -0.3 & 0.3 \end{bmatrix}, \quad \beta^{e} = \begin{bmatrix} 0.3 \\ -0.3 \end{bmatrix}, \quad \beta^{c} = \begin{bmatrix} 0 \\ 0 \end{bmatrix}.$$

These matrices are generated using keyword arguments to the constructor of `parLARMEx`. It is also possible to initialize these with custom matrices or random values by passing `[]`. The variance-covariance of the random effects is built using `b_var` to be

$$G = \begin{bmatrix} 0.03 & 0 & 0 & 0 & 0 & 0 & 0 & 0 \\ 0 & 0.03 & 0 & 0 & 0 & 0 & 0 & 0 \\ 0 & 0 & 0.03 & 0 & 0 & 0 & 0 & 0 \\ 0 & 0 & 0 & 0.03 & 0 & 0 & 0 & 0 \\ 0 & 0 & 0 & 0 & 0.03 & 0 & 0 & 0 \\ 0 & 0 & 0 & 0 & 0 & 0.03 & 0 & 0 \\ 0 & 0 & 0 & 0 & 0 & 0 & 0.03 & 0 \\ 0 & 0 & 0 & 0 & 0 & 0 & 0 & 0.03 \end{bmatrix}$$

This is not the final variance-covariance of the RE, because one needs to control for the stability of the autoregressive process. To this end, we sample a numger of `nSamp = 20000` sets from a multivariate normal distribution of $\text{MVN}(0, G)$ and retain `nL2Max = 100` sets for which the absoloute eigenvalues of $\beta^{ar} + b^{ar}$ are less than one. These are stored in `b_ar`, `b_e`, `b_c`, and the variance-covariance of this latter sample as `b_cov`.

This process is accomplished by calling the function

```
genRE_CS(rng,G,B_AR,maxSbj,nSamp)
```

inside the constructor which updates the fields corresponding to the random effects and their variance-covariance structure. In what follows we import these and other function definitions from the file `helperSim.jl` on GitHub, and then construct the parameters using a replicable RNG:

```
[1]: include("helperSim.jl");
```

```
[2]: rng = MersenneTwister(1984);
     par = parLARMEx(rng=rng);
```

5 GENERATING DATA

We assume that the data generating process has a two-level structure. The multiple observations are made on level I and these are nested in level II units. For EMA data, level I could be the daily observations, as many as `nObsL1`, which are nested in single days with a total number of `nL2`. These characteristics together with the varianc-covariance of noise, `sigma`, initial values for days, `M0`, and the vector of known exogenous factors, `E`, are stored in another `struct`.

```
mutable struct simLARMEx
    nAR::Int                  # number of temporally connected nodes
    nObsL1::Int               # number of observation on level I
    nL2::Int                  # number of units on level II
    sigma::Float64            # variance of noise
    M0::Matrix{Float64}       # initial values each day
    E::Vector{Float64}        # known exogenous factors
end
```

The constructor of this `struct` has the following signature,

```
simLARMEx(;rng=MersenneTwister(), nObsL1=10, nL2=36, sigma=.2,
           M0_max=.5, E=[])
```

and keyword arguments as:

- `rng = MersenneTwister()`: feed `parLARMEx.rng` for consistency and replication, initialized anew by default
- `nObsL1 = 10`: number of observation on level I
- `nL2 = 36`: number of units on level II
- `sigma = .02`: variance of noise
- `M0_max = .5`: determines the amplitude of initial values and exogenous factors, 0.5 to keep trajectories mostly in [-1,1]
- `E = []`: known exogenous factors of size [`nL2` x `nObsL1`], drawn randomly from [`0`, `M0_max`] by default

The following code snippet setts up the configuration of the desired data set. The RNG is carried over to perovide replicablity. Finally, `genData()` gives the simulated data along with the noiseless data as dataframes and the signal-to-noise-ratio (SNR).

[3]:
```
sim = simLARMEx(rng=par.rng);
simData, simData0, SNR = genData(par, sim);
```

[4]:
```
# show(first(simData,5),allcols=true)
latexify(round.(simData[1:5,:],digits=2))
```

[4]:

idL2	tL1	M1	M2	E
1	1	−0.07	0.44	0.27
1	2	−0.03	0.2	0.49
1	3	0.2	0.08	0.19
1	4	0.3	−0.04	0.23
1	5	0.19	−0.17	0.26

The returned values are

- `simData`: the simulated data according to the setup so far
- `simData0`: the simulated data without the noise component used to calculate the SNR

- `SNR`: the ratio of the variance of noiseless data to that of noise

6 TRANSFORMING TO CLASSICAL MIXED-EFFECTS MODEL

By stacking mood values, parameters and covariates for every day separately, and forming the design matrices for fixed and random effects, X and Z, data from a single day takes the following matrix form which is equivalent to a linear mixed effects formulation.

$$\boldsymbol{Y}_i = \boldsymbol{X}_i\boldsymbol{\beta} + \boldsymbol{Z}_i\boldsymbol{b}_i + \boldsymbol{\epsilon}_i.$$

In this formulation for a network of two moods and one external nodes one has

$$\boldsymbol{Y}_i = \begin{bmatrix} m_{1,1} & m_{1,2} & \dots & m_{1,n_i} & m_{2,1} & m_{2,2} & \dots & m_{2,n_i} \end{bmatrix}_i^T,$$

$$\boldsymbol{\beta} = \begin{bmatrix} \beta_{11} & \beta_{12} & \beta_{21} & \beta_{22} & \beta_1^e & \beta_2^e & \beta_1^c & \beta_2^c \end{bmatrix}^T.$$

and

$$\boldsymbol{X}_i = \boldsymbol{Z}_i = \begin{bmatrix} m_{1,0} & m_{2,0} & 0 & 0 & e_1 & 0 & 1 & 0 \\ m_{1,1} & m_{2,1} & 0 & 0 & e_2 & 0 & 1 & 0 \\ \vdots & \vdots & \vdots & \vdots & \vdots & \vdots & \vdots & \vdots \\ m_{1,n_i-1} & m_{2,n_i-1} & 0 & 0 & e_{n_i} & 0 & 1 & 0 \\ 0 & 0 & m_{1,0} & m_{2,0} & 0 & e_1 & 0 & 1 \\ 0 & 0 & m_{1,1} & m_{2,1} & 0 & e_2 & 0 & 1 \\ \vdots & \vdots & \vdots & \vdots & \vdots & \vdots & \vdots & \vdots \\ 0 & 0 & m_{1,n_i-1} & m_{2,n_i-1} & 0 & e_{n_i} & 0 & 1 \end{bmatrix}_i.$$

In its general form, the above equation for k moods, Y_i is a $k(n_i - 1) \times 1$ vector of mood values, β is a $(k^2 + 2k) \times 1$ vector of fixed effects, b_i is a $(k^2 + 2k) \times 1$ vector of random effects, X_i and Z_i are $k(n_i - 1) \times (k^2 + 2k)$ design matrices. The random effects b_i and residuals ϵ_i are assumed to be independent with a multivariate normal (MVN) distribution of

$$\begin{bmatrix} \boldsymbol{b}_i \\ \boldsymbol{\epsilon}_i \end{bmatrix} \sim MVN\left(\begin{bmatrix} \boldsymbol{0} \\ \boldsymbol{0} \end{bmatrix}, \begin{bmatrix} \boldsymbol{G} & \boldsymbol{0} \\ \boldsymbol{0} & \boldsymbol{\Sigma}_i \end{bmatrix} \right).$$

This step is done by the following function which gives another data frame suitable for performing the estimation. Here, it is possible to introduce missing values to the analysis, e.g. `miss = .2` for a 20% of missing values at random. By default, it is assumed that there is no missing values, `miss = 0`. The code is available in `helperSim.jl` on GitHub.

```
prepData2Fit(rawData, idL2, endList, exgList; miss=0)
```

with the following arguments:

- `rawData`: the simulated data as a dataframe

- `idL2`: the column name for level II units, e.g., an id for each day, `"idL2"` here
- `endList`: the list of temporelly connected moods, `["M1","M2"]` here
- `exgList`: the list of exogenous factors together with the constant terms, `["E","C"]` here
- `miss`: the ratio of missing values, defaults to zero

[5]:
```
fitData = prepData2Fit(simData,"idL2",["M1","M2"],["E","C"]);
# show(first(fitData,5),allcols=true)
```

[6]:
```
df = fitData[1:5,:]; df[:,3:end] = round.(df[:,3:end],digits=2);
latexify(df)
```

[6]:

idL2	tL1	M	M11	M12	M21	M22	E1	E2	C1	C2
1	2	−0.03	−0.07	0.44	0	0	0.49	0	1	0
1	3	0.2	−0.03	0.2	0	0	0.19	0	1	0
1	4	0.3	0.2	0.08	0	0	0.23	0	1	0
1	5	0.19	0.3	−0.04	0	0	0.26	0	1	0
1	6	0.65	0.19	−0.17	0	0	0.49	0	1	0

7 SETTING UP THE FORMULA FOR FITTING

The prepared data has more columns representing the response variable `M`, and the predictors including the connections in the network `M11, M12, M21, M22, E1, E2`, as well as the constant terms `C1, C2`. It also contains the level I time points `tL1`, and the level II identifiers `idL2`. We provide a function in `helperSim.jl`, on GitHub,

```
setFormula(fitData)
```

which constructs the formula suitable to feed in the Julia package MixedModels.jl. This function is restricted only to a dataframe which has the exact columns as shown above.

[7]:
```
frm = setFormula(fitData);
```

8 FITTING

We use the MixedModels.jl package in Julia to perform the estimation. It is similar to the lme4 in R but exhibits faster performance in our case.

[8]:
```
fit = MixedModels.fit(MixedModel, frm, fitData, progress=false);
show(fit)
```

```
Linear mixed model fit by maximum likelihood
 M ~ 0 + M11 + M12 + M21 + M22 + E1 + E2 +
    (0 + M11 + M12 + M21 + M22 + E1 + E2 + C1 + C2 | idL2)
   logLik   -2 logLik     AIC        AICc         BIC
```

```
   246.8663  -493.7327  -407.7327  -401.4678  -215.3554

Variance components:
         ColumnVariance Std.Dev.   Corr.
idL2     M11  0.01 0.13
         M12  0.06 0.25 +0.26
         M21  0.02 0.15 -0.50 +0.68
         M22  0.04 0.21 -0.70 +0.01 +0.37
         E1   0.02 0.16 +0.49 +0.27 -0.05 -0.58
         E2   0.02 0.14 +0.77 +0.57 -0.11 -0.45 +0.28
         C1   0.03 0.17 -0.11 +0.20 +0.41 -0.15 +0.38 -0.45
         C2   0.03 0.17 +0.33 +0.03 -0.15 -0.17 -0.31 -0.01 +0.34
Residual      0.017568 0.132545
 Number of obs: 648; levels of grouping factors: 36

  Fixed-effects parameters:
-------------------------------------------
         Coef.  Std. Error      z  Pr(>|z|)
-------------------------------------------
M11   0.16115    0.0441711   3.65    0.0003
M12  -0.482173   0.059415   -8.12    <1e-15
M21  -0.281178   0.0449767  -6.25    <1e-09
M22   0.342208   0.0553473   6.18    <1e-09
E1    0.394389   0.0587841   6.71    <1e-10
E2   -0.254405   0.0542693  -4.69    <1e-05
-------------------------------------------
```

The following parameters have been estimated. It is evident that the network's structure, with regard to the type of connections, has been accurately reconstructed. However, the precision of the coefficient estimations is not optimal.

$$\hat{\beta}^{ar} = \begin{bmatrix} 0.16 & -0.48 \\ -0.28 & 0.34 \end{bmatrix}, \quad \hat{\beta}^{e} = \begin{bmatrix} 0.39 \\ -0.25 \end{bmatrix},$$

9 PARAMETER RECOVERY

In order to assess how well one can recover the parameters, we generate data with the following specifications:

- number of observations per day: 10
- $(\sigma^2, \text{SNR}) \in \{(0.01, 12), (0.02, 6), (0.06, 2)\}$
- number of days, $N \in \{4, 6, \ldots, 36\}$
- β^{ar}, β^{e} and β^{c} as before
- data is generated for one simulated subject 1000 times

- at each iteration new random effects are used

This can be done using the GitHub file, `simBootstrap.jl`. It uses the following function to generate data repeatedly in a loop.

```
loopSim(csvDir, M0_max, sigma, n, P)
```

This function creates a folder called `bootstrap` in the current working directory and for each noise intensity, `sigma`, saves the true and estimated parameters of each simulation in different folders, `re` and `reh`, based on the number of days as `CSV` files. Fixed effects and the variances of random effects are collected in two separate `CSV` files.

The code could take some time, depending on the computational power. Using the estimated parameters one can construct bootstrap confidence intervals for fixed effects and the variance of random effects as well as the relative errors of the estimations. For details regarding these analyses, please refer to the main text.

For an up-to-date version please refer to the GitHub repository. Source code for generating Figures is not provided.

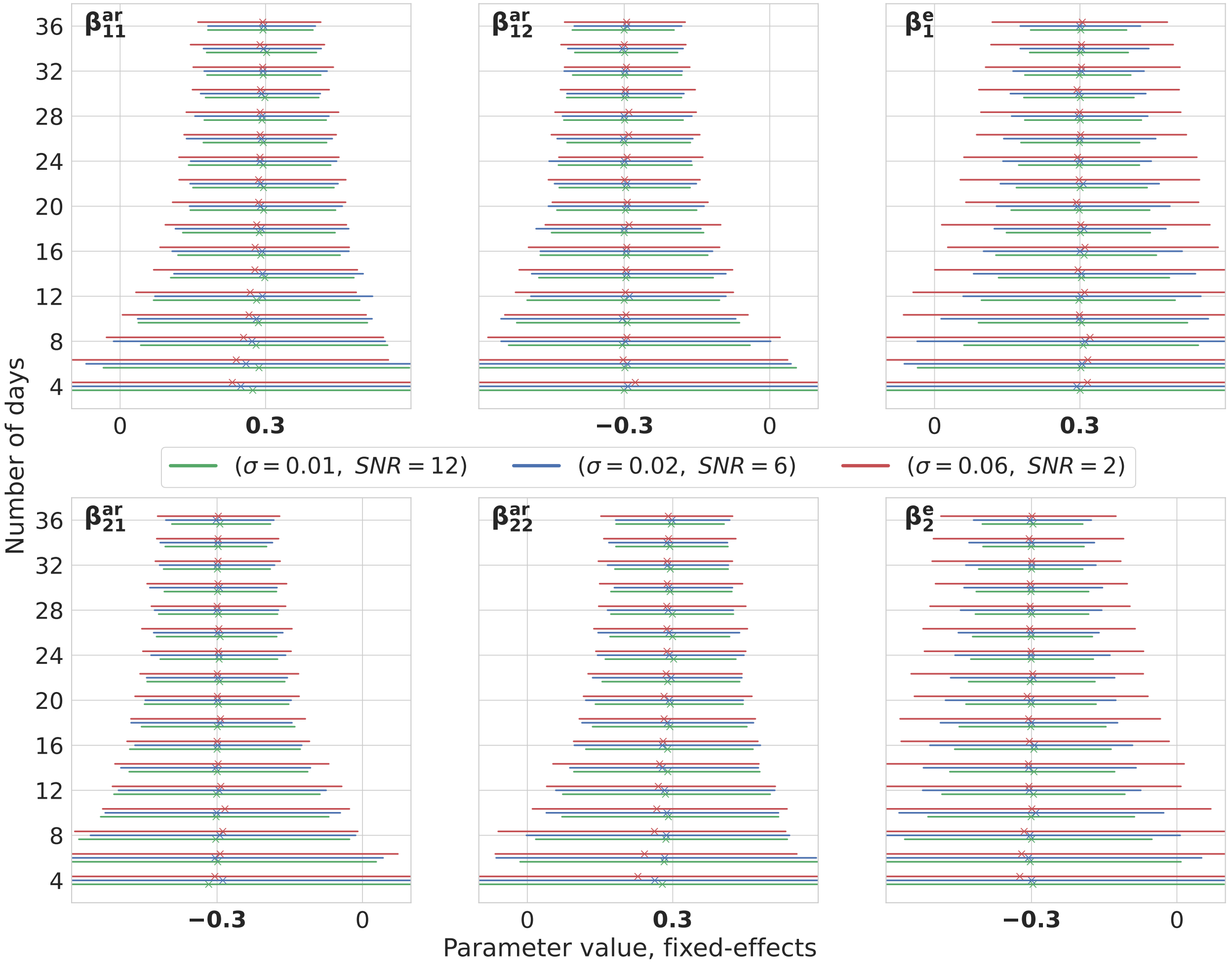

The number of observations per day is 10. True values of parameters are highlighted bold on x axes. For every number of days at the horizontal axes, three lines are drawn representing the bootstrap 95% confidence intervals around the median depicted by cross signs. Every line is color-coded to demonstrate one noise intensity. Data has been generated for one simulated subject, 1000 times repeatedly, with $(\sigma^2, \mathrm{SNR}) \in \{(0.01, 12), (0.02, 6), (0.06, 2)\}$.

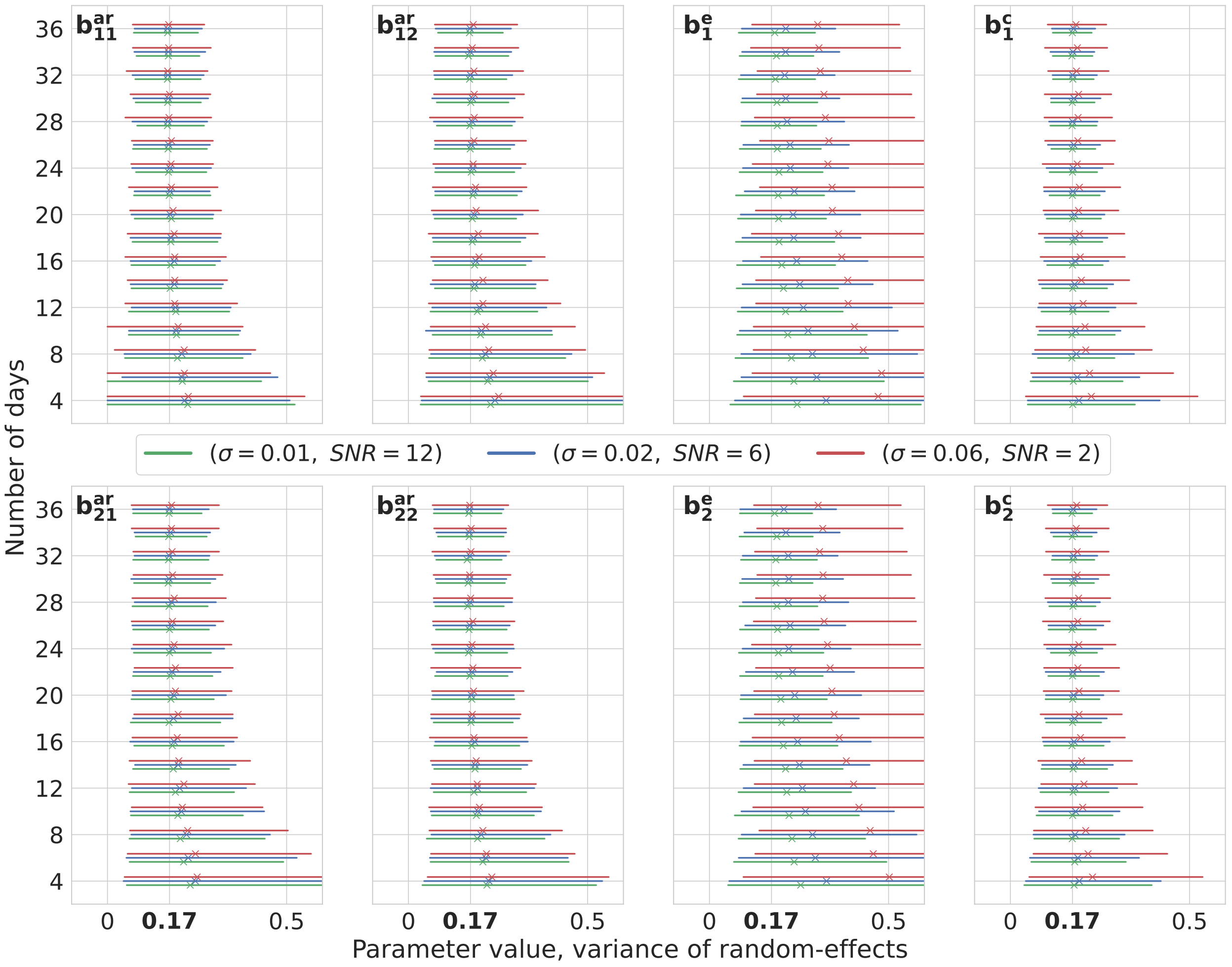

True values of parameters are highlighted bold on x axes, $\sqrt{0.03} \approx 0.17$.

10 FITTING DATA IN R

Given that *R* is widely used in the field, we also provide a graphical user interface as a *Siny Web app* that is available in a separate GitHub repository. It is implemented as an R package to be installed locally by users and is deployed on a server. Without going into further details we refer the interested readers to https://github.com/spooseh/LARMExShiny for the source code. The Web application is accessible through https://spooseh.shinyapps.io/larmexShiny/

11 CONCLUSION

We presented an extension of linear mixed-effects models by adding an autoregressive component to model the network representation of mental state. Specifically, we assumed a simple network of two causally interacting moods under the unidirectional influence of an external-factors node. This framework is suitable for intensive longitudinal data.

We showed briefly how this representation is mathematically formulated and detailed the implementation of such a model in Julia. The practical implementation of data generating process was shown by explicit codes which is available on a public repository under GitHub. We also provided exemplary code for performing a bootstrap analysis to construct confidence intervals for the estimations. The goal here was to calibrate the model through simulations by quantifying the estimation errors. Further work is needed to perform the calibration and validation using real data.

In this text, we mainly discussed the number of observations per subject by adding more days for a specific intensity of noise in data. However, it would be straightforward to modify the provided code to simulate data for other real-world circumstances.

REFERENCES

Epskamp, S. (2020). Psychometric network models from time-series and panel data. *Psychometrika* 85, 206–231

Fitzmaurice, G. M., Laird, N. M., and Ware, J. H. (2011). *Applied Longitudinal Analysis*. Wiley Series in Probability and Statistics (John Wiley & Sons, Inc., Hoboken, NJ), second edn.

Funatogawa, I. and Funatogawa, T. (2018). *Longitudinal Data Analysis: Autoregressive Linear Mixed Effects Models* (Springer Singapore)

Hamilton, J. D. (1994). *Time Series Analysis* (Princeton University Press, Princeton, NJ)

Lütkepohl, H. (2005). *New Introduction to Multiple Time Series Analysis* (Springer-Verlag, Berlin)
